# Association between healthy lifestyle practices and life purpose among a highly health-literate cohort: a cross-sectional study

**DOI:** 10.1186/s12889-021-10905-7

**Published:** 2021-04-29

**Authors:** Nobutaka Hirooka, Takeru Kusano, Shunsuke Kinoshita, Ryutaro Aoyagi, Nakamoto Hidetomo

**Affiliations:** Department of General Internal Medicine, Saitama Medical University, Morohongo 38, Moroyama-machi, Iruma-gun, Saitama, 350-0495 Japan

**Keywords:** Lifestyle, Health-related lifestyle, Purpose in life, Health literacy

## Abstract

**Background:**

The national health promotion program in the twenty-first century Japan (HJ21) correlates life purpose with disease prevention, facilitating the adoption of healthy lifestyles. However, the influence of clustered healthy lifestyle practices on life purpose, within the context of this national health campaign remains uninvestigated. This study assessed the association between such practices and life purpose, in line with the HJ21.

**Methods:**

We performed a nationwide cross-sectional survey on certified specialists in health management. Participants’ demographic information, lifestyle, and purpose in life were measured using a validated tool. The cohort was median-split into two groups based on their clustered health-related lifestyle score. The values for health-related lifestyle and purpose were compared between the two groups and the correlation between health-related lifestyle and purpose in life was measured.

**Results:**

Data from 4820 participants were analyzed. The higher-scoring health-related lifestyle group showed a significantly higher life purpose than the lower group (35.3 vs 31.4; *t* = 23.6, *p* < 0.001). There was a significant association between the scores of clustered healthy lifestyle practices and life purpose (*r* = 0.401, *p* < 0.001). The higher-scoring health-related lifestyle group achieved a higher life purpose than the lower-scoring group. This association between healthy lifestyle practices and life purpose denotes a positive and linear relationship.

**Conclusions:**

Our results suggest that individuals who have a better health-related lifestyle gain a higher sense of life purpose. In other words, a healthy lifestyle predicts a purpose in life. Our findings posit that examining the causal relationship between healthy lifestyle and purpose in life may be a more efficient approach toward health promotion.

## Background

Several studies have investigated the implications of life purpose, and literature has shown that a strong sense of purpose in life was positively associated with positive health outcomes [1–6]. Thus, having a sense of purpose in life is a vital component of human life. Due to a rapidly aging society in Japan, a national health promotion program in the twenty-first century—Health Japan twenty-first century (HJ21)—considers purpose in life as one of the major target goals of health promotion [7].

Purpose in life is defined as “a self-organizing life aim that stimulates goals” [1] and is known to promote healthy behaviors and give life meaning [8, 9]. *Ikigai* is a Japanese word for what is considered an important factor for achieving better health and a fulfilling life [10]. *Ikigai* is defined as something to live for, exemplifying the joy and the goal of living [11]. Although *Ikigai* may not be fully comparable to purpose in life, it does contain the respective concept and plays a cardinal role in yielding positive health-related outcomes [12].

Notably, health outcomes associated with life purpose or *Ikigai* include physical [1, 12, 13] and mental health [3, 13], quality of life [4], disease mortality [1, 12], and longevity [12]. Possessing a strong sense of purpose in life has been associated with a lower risk of mortality and cardiovascular diseases [1] (relative risk: 0.83 and 0.83, respectively). The study concluded that purpose in life tends to yield health benefits. One of the mechanisms considered in the literature was the benefits associated with a healthy lifestyle. People who have adopted a higher purpose in life tend to follow healthier lifestyle practices, which may decrease the incidence of non-communicable chronic diseases, such as cardiovascular diseases or cancer.

Healthcare personnel are responsible for the health of their patients. Studies have shown that healthcare personnel are more likely to encourage healthy lifestyle behaviors among their patients if they engage in such behaviors themselves. Our study population comprises certified specialists in health management who routinely provide advice on health to individuals in their community. Investigating the relationship between lifestyle and purpose in life among healthcare personnel, our target population, is therefore of great scientific interest.

There is a hierarchy of causality among chronic diseases. Non-communicable diseases, such as diabetes, stroke, cancer, and coronary artery disease, have risk factors. In the case of risk factors, such as hypertension, smoking, dyslipidemia, hyperglycemia, studies typically signified proximal causes [14, 15]. A healthy lifestyle is a central causality for these risk factors and thus basic lifestyle should be considered a fundamental and proximal risk factor for the aforementioned non-communicable diseases. Studies also highlight that healthy lifestyle practices prevent many similar chronic diseases [16, 17], and that intervening to promote healthier lifestyle decreases mortality due to non-communicable diseases [18, 19]. Hence, the notion that health benefits are brought through a healthy lifestyle may be supported if the lifestyle strongly correlates with purpose in life.

In this context, however, research exploring the association between purpose in life and healthy lifestyle practices remain scarce. Moreover, existing literature usually considers a single health behavior in relation to purpose in life. To determine the relationship between purpose in life and clustered health-related lifestyle—the fundamental and proximal cause of many health outcomes—the potential benefits of purpose in life towards disease prevention and health must be deciphered.

This study aimed to investigate the association between health-related lifestyles, in line with the HJ21, and purpose in life, measured with a validated tool to better understand the relational mechanisms.

## Method

### Study design

The design was a cross-sectional study on a cohort of nationwide certified specialists in health management. We surveyed health-related lifestyles similar to those in the questionnaire used for the HJ21. Our questionnaire is based on the one of the oldest national health surveys around the world, the National Health and Nutrition Survey conducted by Japanese Government [20]. This survey is the oldest of all national health examination surveys currently conducted worldwide and serves as a comprehensive database for risk factors related to non-communicable diseases in Japan. The survey includes questions on demographic data and health-related habits, such as physical activity and exercise, nutrition and diet, smoking, stress, and alcohol intake. Purpose in life was measured with a validated tool in Japanese using the purposeful life scale (Ikigai-9) [21]. The ethics committee of the Saitama Medical University approved the study (ID: 896, 2018).

### Participants

Study participants were certified specialists in health management who actively pursued professional growth provided by the Japanese Association of Preventive Medicine for Adult Disease [22]. This certification is sponsored by the Ministry of Education, Culture, Sports, Science and Technology, Japan. We excluded specialists who did not actively engage in continuing education or health promotion activities. These specialists are expected to engage the community and the society they live in to promote health and wellbeing. Specialists in health management are certified in multiple processes of study. Candidates study various aspects within the course, including health promotion, lifestyle-related diseases, mental health, nutrition, environment and health, physical activity and exercise, emergency medicine, life support, and health care system. To register, candidates must pass the final written examination. The Japanese Association of Preventive Medicine for Adult Disease encourages specialists to participate in numerous activities by facilitating health promotion workshops, speeches, and activities after registration. Among these individuals who met our inclusion criteria (*N* = 9149), 4820 agreed to participate in the survey.

### Variables and measurements

Variables measured in this study were demographic characteristics; health-related habits, including physical activity and exercise, nutrition and diet, smoking, stress, and alcohol intake; and purpose in life. There were eleven health-related lifestyle questions, of which five were two-scaled (“Intention to maintain ideal weight,” “Exercise,” “Alcohol intake,” “Manage lifestyle to prevent disease,” and “Smoking”). For these items, a score of “1” was assigned for an unhealthy lifestyle and a score of “4” was assigned for a healthy lifestyle. The rest of the six health-related habits (“Reading nutritional information labels,” “Maintaining a balanced diet in daily life,” “Intention for exercise,” “Stress,” “Rest,” and “Sleep”) were to be answered on a four-point scale, from “4” (most favorable) to “1” (least favorable). Finally, we added the values of each answer to the questions on the health-related lifestyle of the participants as their clustered health-related lifestyle scores. To measure purpose in life, we used the Ikigai-9 scale, a validated tool to quantify purpose in life. The Ikigai-9 is a psychometric tool that measures across the dimensions of (1) optimistic and positive emotions toward life, (2) active and positive attitudes towards one’s life, and (3) acknowledgement of the meaning of one’s existence [23]. The Ikigai-9 scale consists of nine questions on various aspects of life purpose and each question must be answered on a five-point scale, from “1” (Strongly disagree) to “5” (Strongly agree). These variables and measurements were previously described elsewhere [24]. Considering the variables, age, weight, height, BMI, volume of alcohol intake, and purpose in life scores were numeric. Sex, healthy lifestyle, smoking, alcohol intake, and stress comprised either binary or ordinal data.

### Analysis

Descriptive statistics (i.e., mean, standard deviation, range) were used to describe participants’ characteristics. The cohort was divided into two groups (i.e., a higher and lower group, with a cut-off using the median score) based on the clustered health-related lifestyle scores. The correlations between age and lifestyle score and between age and purpose in life score were analyzed. The difference in the Ikigai-9 score between the two clustered health-related lifestyle score groups was investigated. Further, the effect size of the difference in Ikigai-9 score between the two groups was calculated with using Cohen’s *d*. The association between the clustered health-related lifestyle score and the Ikigai-9 score was also analyzed as a bivariate correlation and a correlation coefficient was calculated to see whether the health-related lifestyles accounted for life purpose. A multiple regression analysis was performed to determine the association between the clustered health-related lifestyle score and the purpose in life score, after controlling for age. All statistical tests were two-tailed and the software IBM SPSS Statistics (Version 26.0. Armonk, NY) was used for the analysis.

## Results

The demographic and health-related lifestyle characteristics of the study participants are shown in Table 1. In total, 4820 certified specialists in health management were included in the analysis. There were 3190 women (66.2%) and 1630 men (33.8%). The mean (*SD*) age of all study participants was 55.4 (±12.2) years. The majority of the participants (85.0%) were non-obese and “intended to keep ideal weight” and “maintain a healthy lifestyle (82.6% and 89.2%, respectively) to prevent lifestyle-related disease,” such as obesity, metabolic syndrome, and cardiovascular disease. We also found that more than 80% of the study participants “read nutritional information labels” and more than 90% “maintained a balanced diet in daily life.” Regarding exercise and physical activity, more than 80% of the study participants “intended to exercise” and approximately 64% of them achieved the recommended levels. These findings reflected a low rate of obesity among the participants, which was 15.0% in the study. While most of the participants reported resting and sleeping adequately, the rate of taking on stress was high (74.4%). The descriptive analysis of the Ikigai-9 scores confirmed that it was normally distributed, based on the histogram and P-P plot.

**Table 1:** Demographic Characteristics of the Cohort

Characteristics	Total
Sex	
Male	1630
Female	3190
Age range	4820
< 30 years	129 (2.7)
30–39 years	372 (7.7)
40–49 years	930 (19.3)
50–59 years	1541 (32.0)
60–69 years	1291 (26.8)
70–79 years	489 (10.1)
≥ 80 years	68 (1.4)
Age (Ave years, *SD*)	55.4 (12.2)
Height (Ave cm, *SD*)	161.3 (8.0)
Weight (Ave kg, *SD*)	57.5 (10.8)
BMI (Ave kg/m^2^, *SD*)	21.9 (3.3)
Obesity (%)	15.0
Intention to keep ideal weight (%)	
Yes	82.6
No	17.4
Managing Lifestyle for disease prevention (%)	
Yes	89.2
No	10.8
Reading nutritional information labels (%)	
Always	34.3
Often	47.9
Rarely	13.4
Very rarely	4.3
Maintaining a balanced diet in daily life (%)	
Always	52.8
Often	38.0
Rarely	8.0
Very rarely	1.2
Intention for exercise (%)	
Always	42.3
Sometimes	41.3
In the past	13.2
Never	3.1
Adequate Exercise (%)	
Yes	63.9
No	36.1
Excessive alcohol intake (%)	5.8
Smoking (%)	
Current	6.1
Past	18.0
None	75.8
Stress (%)	
High	20.4
Moderate	54
Low	21.8
None	3.7
Rest (%)	
Satisfactory	20.5
Adequate	54.0
Not adequate	21.8
Not satisfactory	3.7
Sleep (%)	
Satisfactory	21.3
Adequate	57.4
Not adequate	20.3
Not satisfactory	1.0

Table 2 shows the demographics and healthy lifestyle practices for both the higher and lower clustered health-related lifestyle score groups. We found consistent favorable results in all measured health-related habits in the higher clustered health-related lifestyle score group. There was a significant difference in the scores of purpose in life between the higher group and the lower clustered health-related lifestyle score group (*t* = 23.6, *p* < .0001). In the higher group, the average score of purpose in life was 35.3 (95% CI; [35.1–35.5]), while for the lower group, the average score for purpose in life was 31.4 (95% CI; [31.2–31.7]). The differences in the Ikigai-9 purpose in life scores of the two groups and its effect sizes (Cohen’s d) were 3.8 (95% CI; [3.5–4.2]) and 0.68, respectively. Moreover, there was a significant association between the clustered health-related lifestyle score and purpose in life score, *r* = .401, *p* < .001. The significance remained after controlling for age. Correlation between age and both lifestyle and purpose in life were significant (Pearson *r* = 0.29 and 0.15, respectively; both *p* < .05).

**Table 2:** Comparison between Lower and Higher Health-related Lifestyle Groups

Characteristics	High	Low
Sex		
Male	882	748
Female	1701	1489
Age (Ave years, SD)	58.2 (12.0)	52.1 (11.5)
BMI (Ave kg/m^2^, SD)	21.8 (3.0)	22.0 (3.7)
Obesity (%)	12.2	18.3
Intention to keep ideal weight (%)		
Yes	92.8	70.3
Managing Lifestyle for disease prevention (%)		
Yes	94.4	82.7
Reading nutritional information labels (%)		
Always	44.7	22.2
Often	45.4	50.8
Rarely	7.8	19.9
Very rarely	1.9	7.0
Maintaining a balanced diet in daily life (%)		
Always	68.3	34.9
Often	29.6	47.6
Rarely	2.0	14.9
Very rarely	0.1	2.5
Intention for exercise (%)		
Always	63.6	17.6
Sometimes	33.6	50.3
In the past	2.6	25.5
Never	0.2	6.5
Adequate Exercise (%)		
Yes	90.6	33.0
Excessive alcohol intake (%)	2.7	9.3
Smoking (%)		
Current	17.6	18.5
Past	1.2	11.6
None	81.0	69.1
Stress (%)		
High	10.8	31.6
Moderate	54.4	53.6
Low	28.6	13.9
None	6.3	0.8
Rest (%)		
Satisfactory	32.5	8.5
Adequate	55.1	53.0
Not adequate	11.8	32.0
Not satisfactory	0.5	6.5
Sleep (%)		
Satisfactory	32.1	8.8
Adequate	56.6	58.2
Not adequate	11.1	30.9
Not satisfactory	0.1	2.1
Purpose in life score ([95% CI])	35.3 (35.1–35.5)	31.4 (31.2–31.7)

## Discussion

We found that the higher-scoring clustered health-related lifestyle group showed a statistically significant higher purpose in life than the lower-scoring clustered health-related lifestyle group. The study also highlighted a significant positive association between the clustered health-related lifestyle score and the Ikigai-9 score. To the best of our knowledge, this study was the first to show that a strong sense of purpose in life correlates with clustered health-related lifestyles in the context of a national health campaign. Several studies indicate a positive relationship between purpose in life and health-related lifestyles [1, 25–27]. Furthermore, many publications reveal a correlation between a single healthy habit and purpose in life. Therefore, our findings—that affirm a positive relationship between purpose in life and clustered health-related lifestyle—are consistent with previously reported results and help broaden the evidence of this association.

Exploring the mechanistic link of purpose in life with a healthy lifestyle may help us understand this relationship. While studies highlight the positive relationship between purpose in life and health-related lifestyle, a few studies’ results are inconsistent with our findings. For example, an existing prospective study did not observe a positive association between purpose in life and healthy sleep patterns [28]. In other studies, the purpose of life was not associated with smoking [29, 30]. Notably, the mechanistic link between health-related lifestyle and purpose in life has not been well examined. Hooker et al. proposed a hypothesized model linking purpose in life with health [31]. They summarized the relationship between life purpose and health outcomes by utilizing the concept of self-regulation. In the model, they proposed that life purpose influenced health through three self-regulatory processes and skills: stress-buffering, adaptive coping, and health behaviors. Health-related lifestyle, one of the self-regulatory processes, is the result of individuals setting goals, monitoring their progress, and using feedback to modify their lifestyle [31]. Thus, a purpose provides the foundation and motivation for engaging in a healthy lifestyle. Kim et al. also suggested that sense of purpose in life enhances the likelihood for engagement in restorative health-related lifestyle practices (e.g., physical activity, healthy sleep quality, use of preventive health care services) from cardiovascular disease to the indirect effect of behavior [32].

There is an alternative explanation for the mechanistic link between purpose in life and health-related lifestyle. A reverse causality model suggested that engaging in healthy lifestyle practices could predict a greater purpose in life [31, 33]. Our results denoted that the group with a higher score in purpose in life performed healthier lifestyle practices and behaviors (Table 2), which can be supported by either of the hypothesized models. Age statistically significantly influenced both lifestyle and purpose in life in this study, while gender did not. However, age did not change overall relation between lifestyle and purpose in life. This infers that age may act as a moderator on the association. Further research is needed to clarify the mechanism and the directionality of the association, including any modifying factors. The mechanism to explain the causal relationship between life purpose and healthy lifestyle practices helped prepare for healthy aging by preventing diseases, increasing health longevity, and imbuing a health-oriented drive, which are the major goals of the HJ21.

Additionally, the difference in life purpose scores between the two groups (35.3 vs 31.4), shown in Table 2, should be further explored, whilst we found a statistically significant difference and a correlation between healthy lifestyle practices and purpose in life. Rather than being a single concept, purpose in life has several elements and a more comprehensive construct. The majority of measurement tools concerned with meaning in life assess two distinct concepts: coherence and purpose [34]. Coherence is a sense of comprehensibility, or one’s life “making sense,” which is descriptive and value-neutral. Purpose means a sense of core goals, aims, and direction in one’s life, which is more evaluative and value-laden in concept. *Ikigai* is the Japanese concept meaning a sense of life worth living. The Ikigai-9 scale used in this study has three constructs for measuring the purpose in life; (1) optimistic and positive emotions toward life, (2) active and positive attitudes towards one’s life, and (3) acknowledgement of the meaning of one’s existence. The scale seems to measure more similarly to the purpose; however, the total score does not distinguish between the association of specific constructs and healthy lifestyle practices. Thus, further methodological sophistication regarding the evaluation of a specific concept encompassed within life purpose needs to be reached. This aspect broadens our understanding of purpose in life and its relation to health. This particular cohort of certified specialists shared many features of high health literacy through the process of professional development and certification, combined with life-long learning and activities related to their role as health management specialists. Further, health-related lifestyle practices mean that the certified specialists were far healthier than the national average. These characteristics represent an individual’s health literacy. Health literacy is considered to be an individuals’ capacity to obtain and understand basic health information and services and to make appropriate health-related decisions based on this information [35]. Therefore, health literacy is directly associated with disease mortality [36], overall health status [37], disease prevention [38, 39], and health behaviors. These can be attributed to purpose in life [2].

Thus, health literacy and health-related lifestyle appear to have a similar relationship with disease prevention and better health outcomes. The mediating effect of health literacy on the relationship between healthy lifestyle and life purpose should be investigated. Such inquiries in a prospective cohort study can better explain the mechanism of the causal link between purpose in life, health-related lifestyle, and health literacy.

### Limitations

There are several limitations to this study. First, all the measurements were self-reported, which can be a source of bias. Second, while the survey questionnaires are widely used in national health promotion, they have not been fully validated. Third, the real-life meaning of purpose in life has not been determined yet. The Ikigai-9 score, one of the tools used to measure the life purpose score, was validated in a small and limited population; however, the instrument may not capture it holistically. This limitation was implicated by the previously reported systematic review. Furthermore, Zheng et al. found variability in the strength of correlation among the questionnaire for quality of life, part of which included questions regarding a purposeful life [40]. Lastly, the correlational analysis did not include an adjustment for confounding factors other than age. Hence, little is known about factors influencing the relationship between a healthy lifestyle and purpose in life. We need to establish other potential influencing factors and determine which variables have mediating, moderating, and confounding effects on purpose in life to understand the causal relationship between healthy lifestyle practices and life purpose [41]. This exploration proposes a promising model for future intervention programs.

### Strengths

Despite these limitations, this study has several strengths. First, the study sample size, *N* = 4820, was large and distributed throughout Japan. This aspect of the study increases generalizability. According to the previous review, numerous studies on purpose in life focused on older adults [42], whereas only a few were concerned with younger or middle-aged adults. In the present study, the majority of the participants were younger and middle-aged adults. Second, previous studies used relatively simple questions or did not employ validated tools to measure purpose in life. However, we used a validated tool, Ikigai-9, in this study. This aspect allows the study results to increase the reliability and validity of the measurement of purpose in life and also hold applicability in other studies. Lastly, study participants were certified specialists in health management who have shown high health literacy. This inclusion criterion provides guidance on improving healthy lifestyle practices through health literacy as an approach to health promotion.

## Conclusions

In conclusion, a healthy lifestyle was found to be positively associated with purpose in life among a cohort of highly health-literate professionals. Healthcare personnel who receive specific training for health management may play important roles in promoting a population’s health and wellbeing. However, the mechanism to explain the relationship between purpose in life and health-related lifestyle remains unknown. Therefore, causal relations between improving healthier lifestyles and increasing purpose in life should be tested.

## Data Availability

The datasets used in the current study are available from the corresponding author upon reasonable request.

## References

[1] Cohen R, Bavishi C, Rozanski A (2016). Purpose in life and its relationship to all-cause mortality and cardiovascular events: a meta-analysis. Psychosom Med.

[2] Roepke AM, Jayawickreme E, Riffle QM (2014). Meaning and health: a systematic review. Appl Res Qual of Life.

[3] Wood AM, Joseph S (2010). The absence of positive psychological (eudemonic) well-being as a risk factor for depression: a ten year cohort study. J Affect Disord.

[4] Park CL, Malone MR, Suresh DP, Bliss D, Rosen RI (2008). Coping, meaning in life, and quality of life in congestive heart failure patients. Qual Life Res.

[5] Sherman AC, Simonton S (2012). Effects of personal meaning among patients in primary and specialized care: associations with psychosocial and physical outcomes. Psychol Health.

[6] Kraus N (2009). Meaning in life and mortality. J Gerontol B, Psycol Sci Soc Sci.

[7] Ministry of Health, Labour, and Welfare. Health Japan 21, 2^nd^ phase. https://www.mhlw.go.jp/file/06-Seisakujouhou-10900000-Kenkoukyoku/0000047330.pdf. Accessed 31 October 2020.

[8] McKnight PE, Kashdan TB (2009). Purpose in life as a system creates and sustains health and well-being: an integrative, testable theory. Rev Gen Psychol.

[9] Ryff CD, Keyes CM (1995). The structure of psychological well-being revisited. J Pers Soc Psychol.

[10] Nakanishi N (1999). “Ikigai” in older Japanese people. Age Ageing.

[11] Sone T, Nakaya N, Ohmori K, Shimazu T, Higashiguchi M, Kakizaki M, Kikuchi N, Kuriyama S, Tsuji I (2008). Sense of life worth living (Ikigai) and mortality in Japan: Ohsaki study. Psychosom Med.

[12] Tanno K, Sakata K, Ohsawa M, Onoda T, Itai K, Yaegashi Y, Tamakoshi A, JACC Study Group (2009). Association of Ikigai as a positive psychological factor with all-cause mortality and cause-specific mortality among middle-aged and elderly Japanese people: findings from the Japan collaborative cohort study. J Psychosom Res.

[13] Nakanishi N, Fukuda H, Tatara K (2003). Changes in psychosocial conditions and eventual mortality in community-residing elderly people. J Epidemiol.

[14] Mozaffarian D, Wilson PF, Kannel WB (2008). Beyond established and novel risk factors: lifestyle factors for cardiovascular disease. Circulation..

[15] Egger G, Dixon J. Beyond obesity and lifestyle: a review of 21st century chronic disease determinants. Biomed Res Int. 2014;2014:731685. 10.1155/2014/731685 PMC3997940 24804239

[16] Cecchini M, Sassi F, Lauer JA, Lee YY, Guajardo-Barron V, Chisholm D (2010). Chronic diseases: chronic diseases and development 3 tackling of unhealthy diets, physical inactivity, and obesity: health effects and cost-effectiveness. Lancet..

[17] Bodai BI, Nakata TE, Wong WT, Clark DR, Lawenda S, Tsou C, Liu R, Shiue L, Cooper N, Rehbein M, Ha BP, Mckeirnan A, Misquitta R, Vij P, Klonecke A, Mejia CS, Dionysian E, Hashmi S, Greger M, Stoll S, Campbell TM (2018). Lifestyle medicine: a brief review of its dramatic impact on health and survival. Perm J.

[18] Anderson E, Durstine JL (2019). Physical activity, exercise, and chronic diseases: a brief review. Sports Med Health Sci.

[19] Achieving global targets: healthy lifestyles to prevent and control non-communicable diseases. World Health Organization, 2013 https://www.who.int/china/news/detail/12-11-2013-achieving-global-targets-healthy-lifestyles-to-prevent-and-control-non-communicable-diseases. Accessed 31 October 2020.

[20] Katanoda K, Matsumura Y (2002). National Nutrition Survey in Japan—its methodological transition and current findings. J Nutr Sci Vitaminol.

[21] Imai T, Osada H, Nishimura Y (2012). The reliability and validity of a new scale for measuring the concept of Ikigai (Ikigai-9). Nihon Koshu Eisei Zassi.

[22] Japanese Association of Preventive Medicine for Adult Disease. Kenko kanrisi [Specialists in health management]. https://www.healthcare.or.jp. Accessed 31 October 2020).

[23] Fido D, Kotera Y, Asano K (2020). English translation and validation of the Ikigai-9 in a UK sample. Int J Ment Health Addict.

[24] Kinoshita S, Hirooka N, Kusano T, Saito K, Nakamoto H (2020). Does improvement in health-related lifestyle habits increase purpose in life among a health literate cohort?. Int J Environ Res Pub Health.

[25] Morimoto Y, Yamasaki S, Ando S, Koike S, Fujikawa S, Kanata S, Endo K, Nakanishi M, Hatch SL, Richards M, Kasai K, Hiraiwa-Hasegawa M, Nishida A (2018). Purpose in life and tobacco use among community-dwelling mothers of early adolescents. BMJ Open.

[26] Hooker SA, Masters KS (2016). Purpose in life is associated with physical activity measured by accelerometer. J Health Psychol.

[27] Conner TS, Brookie KL, Richardson AC, Polak MA (2015). On carrots and curiosity: eating fruit and vegetables is associated with greater flourishing in daily life. Br J Health Psychol.

[28] Ryff CD, Singer B, Love GD (2004). Positive health: connecting well-being with biology. Philos Trans R Soc Lond Ser B Biol Sci.

[29] Steptoe A, Fancourt D (2019). Leading a meaningful life at older ages and its relation with social engagement, prosperity, health, biology, and time use. Proc Natl Acad Sci U S A.

[30] Chen Y, Kim ES, Koh HK, Fraizer AL, Van der Weele TJ (2019). Sense of mission and subsequent health and well-being among young adults: an outcome-wide analysis. Am J Epidemiol.

[31] Hooker SA, Master KS, Park CL (2018). A meaningful life is a healthy life: a conceptual model liking meaning and meaning salience to health. Rev Gen Psychol.

[32] Kim ES, Delaney SW, Kubzansky LD (2019). Sense of purpose in life and cardiovascular disease: underlying mechanisms and future directions. Cur Cardiol Rep.

[33] Kahana E, Lawrence RH, Kahana B, Kercher K, Wisniewski A, Stoller E, Tobin J, Stange K (2002). Long-term impact of preventive proactivity on the quality of life of the old-old. Psychosom Med.

[34] Martela F, Sterger MF (2016). The three meanings of meaning in life: distinguishing coherence, purpose, and significance. J Posit Psychol.

[35] Nutbeam D (2000). Health literacy as a public goal: a challenge for contemporary health education and communication strategies into the 21^st^ century. Health Prom Int.

[36] Baker DW, Wolf MS, Feinglass J, Thompson JA, Gazmararian JA, Huang J (2007). Health literacy and mortality among elderly persons. Arch Intern Med.

[37] Berkman ND, Sheridan SL, Donahue KE, Halpern DJ, Crotty K (2011). Low health literacy and health outcomes: an updated systematic review. Ann Intern Med.

[38] Fernandez DM, Larson JL, Zikmund-Fisher BJ (2016). Associations between health literacy and preventive health behaviors among older adults: findings from the health and retirement study. BMC Public Health.

[39] Santos P, Sá L, Couto L, Hespanhol A (2017). Health literacy as a key for effective preventive medicine. Cogent Soc Sci.

[40] Zheng M, Jin H, Shi N, Duan C, Wang D, Yu X (2018). The relationship between health literacy and quality of life: a systematic review and meta-analysis. Health Qual Life Outcomes.

[41] Alimujiang A, Wiensch A, Boss J, Fleischer NL, Mondul AM, McLean K, Mukherjee B, Pearce CL (2019). Association between life purpose and mortality among US adults older than 50 years. JAMA Netw Open.

[42] Hill PL, Turiano NA (2014). Purpose in life as a predictor of mortality across adulthood. Psychol Sci.

